# LABEL: Fast and Accurate Lineage Assignment with Assessment of H5N1 and H9N2 Influenza A Hemagglutinins

**DOI:** 10.1371/journal.pone.0086921

**Published:** 2014-01-23

**Authors:** Samuel S. Shepard, C. Todd Davis, Justin Bahl, Pierre Rivailler, Ian A. York, Ruben O. Donis

**Affiliations:** 1 Influenza Division, Centers for Disease Control and Prevention, Atlanta, Georgia, United States of America; 2 Laboratory of Virus Evolution in Program of Emerging Infectious Diseases, Duke-NUS Graduate Medical School, Singapore, Singapore; 3 Center for Infectious Diseases, The University of Texas School of Public Health, Houston, Texas, United States of America; The University of Hong Kong, China

## Abstract

The evolutionary classification of influenza genes into lineages is a first step in understanding their molecular epidemiology and can inform the subsequent implementation of control measures. We introduce a novel approach called Lineage Assignment By Extended Learning (LABEL) to rapidly determine cladistic information for any number of genes without the need for time-consuming sequence alignment, phylogenetic tree construction, or manual annotation. Instead, LABEL relies on hidden Markov model profiles and support vector machine training to hierarchically classify gene sequences by their similarity to pre-defined lineages. We assessed LABEL by analyzing the annotated hemagglutinin genes of highly pathogenic (H5N1) and low pathogenicity (H9N2) avian influenza A viruses. Using the WHO/FAO/OIE H5N1 evolution working group nomenclature, the LABEL pipeline quickly and accurately identified the H5 lineages of uncharacterized sequences. Moreover, we developed an updated clade nomenclature for the H9 hemagglutinin gene and show a similarly fast and reliable phylogenetic assessment with LABEL. While this study was focused on hemagglutinin sequences, LABEL could be applied to the analysis of any gene and shows great potential to guide molecular epidemiology activities, accelerate database annotation, and provide a data sorting tool for other large-scale bioinformatic studies.

## Introduction

Influenza A viruses are widespread and diverse within mammals and birds. As with other RNA viruses, they undergo frequent mutations allowing rapid evolution in response to natural selection [1], [2]. The enormous health impact of influenza viruses in humans and animals, and the potentially catastrophic effects of influenza pandemics, have led to large-scale surveillance of mammalian and avian influenza viruses with thousands of gene sequences being generated each year for molecular characterization [3]–[5]. Protection against influenza viruses depends mainly on vaccination and naturally acquired immunity, but the rapid antigenic evolution of these viruses allows them to escape population immunity [6]. Phylogenetic analyses, particularly those based on hemagglutinin (HA) genes, can help characterize groups of related viruses into clades and lineages expected to share common immunologic and/or phenotypic features [7]–[9].

H5N1 and H9N2 are two avian influenza A virus subtypes with significant pandemic potential. Both subtypes have widespread geographic distribution in domestic poultry and have caused occasional disease in humans. Since its identification in China in 1996, descendants of the A/goose/Guangdong/1/1996-like (Gs/GD-like) hemagglutinin gene of highly pathogenic H5N1 have spread across Asia, Africa, and Europe into over 63 countries [10], [11]. While these viruses are not readily transmissible between humans, the case fatality ratio is approximately 58%, with over 600 laboratory-confirmed human infections [12]. By contrast, low pathogenicity H9N2 viruses have been detected infrequently in humans with mild influenza-like illness. Nonetheless, H9N2 continues to cause disease outbreaks throughout much of the world's poultry populations. In recent years, human zoonotic infections that have been reported coincide with increased detection of these viruses in domestic poultry throughout Asia and the Middle East [13].

Both H5N1 and H9N2 viruses have persisted in domestic birds for many years with viral diversification being driven by pronounced spread during outbreaks, continuous interspecies transmission in avian hosts, geographic isolation, and genetic selection—all resulting in the emergence of multiple genetic lineages [14], [15]. H5N1 viruses in particular have been grouped into over 30 genetic clades by the WHO/OIE/FAO H5N1 Evolution Working Group since its classification system was put into place less than ten years ago [5], [16]–[18].

The nomenclature recommendations and ongoing clade determinations for H5N1 are based on phylogenetic analyses and quantification of clade sequence divergence (WHO/OIE/FAO 2008). As such, accurate assignment of new sequences requires the use of the appropriate annotated guide tree (www.who.int/influenza/gisrs_laboratory/h5n1_nomenclature) along with careful application of the WHO/OIE/FAO H5N1 Evolution Working Group guidelines. For large datasets the clade determination process can be time-consuming, requiring the alignment of query and reference sequences, manual correction of alignments, phylogenetic tree construction with sufficient bootstraps, and, finally, pairwise genetic distance calculations.

A number of automated, sequence comparison methods have been developed for lineage assignment, subtyping, and genotyping. BLAST-based methods [19] are fast but are vulnerable to new sequences that have diverged from the reference library. Phylogenetic tree based methods—such as the “two-time test” for genotyping described in [20] where viruses from a recent time window must be clustered with those from an earlier time window—are highly accurate but require computationally-intensive multiple sequence alignment, as well as tree construction, to identify each gene lineage. Composition based approaches, such as Chaos Game Representation, have been shown to effectively identify HIV-1 subtypes with increasing efficacy for whole genomes in comparison to sub-genomic regions [21]. However, their discriminatory power may be limited for the analysis of viruses with segmented genomes, such as influenza, where lineage assignment is done on relatively small gene segments and where clades can have very similar nucleotide composition.

Herein we provide a new method and pipeline for the automated clade annotation of influenza hemagglutinin sequences. The new tool, termed “Lineage assignment by extended learning” (LABEL), can be trained to characterize lineages with broad diversity (e.g., HA subtypes), minor differences (e.g., emerging HA sub-clades) or both, provided the initial lineages are pre-defined. LABEL uses profile hidden Markov models (pHMM) to analyze sequence similarity to various clades and extends the results to support vector machines (SVM) for making lineage assignment decisions. Profile HMMs have found use in remote homolog identification and the determination of protein family membership [22]–[24]. SVMs have been used previously in metagenomics, splice-site recognition, gene finding, and sequence classification [25]–[28].

LABEL was developed, validated and optimized using two influenza A virus HA gene subtypes: highly pathogenic H5N1 and low pathogenicity H9N2 avian influenza viruses. We show excellent accuracy for full-length hemagglutinin gene analysis and fast runtime compared to the usual phylogenetic tree methods. Furthermore, we demonstrate how HMM profile scores can be used to visualize clustering patterns for the annotation of sequences that fail to cluster consistently using traditional phylogenetic analyses. The use of LABEL to rapidly and accurately assign new influenza virus sequences into lineages will aid viral surveillance and disease control activities as well as advance research into finding new clade-specific phenotypes.

## Methods

### Datasets

Nucleotide sequences used in our analyses may be obtained from GISAID (www.gisaid.org), Genbank (www.ncbi.nlm.nih.gov/genomes/FLU), and the WHO website (www.who.int/influenza/gisrs_laboratory/h5n1_nomenclature). GISAID acknowledgement tables for laboratory contributions for both H5N1 and H9N2 hemagglutinins can be found in Supplemental Files S1 and S2. The Supplemental Files also contain a listing of virus strain names, accession numbers, data sources, as well as known annotations versus LABEL predicted annotations for all data used in this study. H5N1 clade annotations were obtained courtesy of *WHO/OIE/FAO H5N1 Evolution Working Group* members [5]. Thanks to advice from working group members and insights gained from our analyses, a few annotations were corrected or updated with respect to the published WHO/OIE/FAO tree datasets, see Supplementary File S1. We excluded laboratory-derived viruses and sequences shorter than 1200 base-pairs. This threshold was more inclusive than the 1,600 nucleotide cutoff used in [5] but still larger than a typical mature HA1 segment (∼960 nts). Collectively, highly pathogenic H5N1 influenza A viruses that share common ancestry with the first isolate (A/goose/Guangdong/1/96) are known as Gs/GD-like while non-Gs/GD viruses include Eurasian and North American low pathogenicity H5 isolates. Multiple sequence entries (i.e., duplicate virus names) and HA sequences with 100% sequence identity were removed using a custom Perl script. For H5 annotation, 2506 sequences were used to create the profile HMMs with 586 of these being further used to train the SVMs. A simplified example of pHMM and SVM training data is shown diagrammatically in Supplemental Fig. S1, steps 2 & 3. SVM training sequences were then removed to test the SVMs. In order to test both the SVMs and pHMMs together, 373 newly submitted GISAID H5 hemagglutinin sequences (1 Feb 2011 to 1 Apr 2012) that satisfied our criteria and were not redundant with any training data were tested. In order to account for H5 lineages outside of the WHO nomenclature studies we used 524 non-Gs/GD lineage H5 HA to create profile HMMs, with 59 being used to train SVMs. We again removed SVM training sequences to test non-Gs/GD H5s, leaving 465 sequences. For H9 annotation, 1592 sequences were used to create HMM profiles, with 342 of them used for SVMs. These were removed to test the SVMs. Most H9 hemagglutinin sequences were from the H9N2 subtype viruses, although H9s paired with other neuraminidase subtypes were also included. For the analysis of partial sequences (fragments) of both H5 and H9 hemagglutinins, shortened sequences were removed if they were more than 5% shorter than the alignment length (ensuring coverage of the region of interest) or if they were redundant with existing sequences in the set.

### Lineage assignment by extending learning

**Figure 1:**
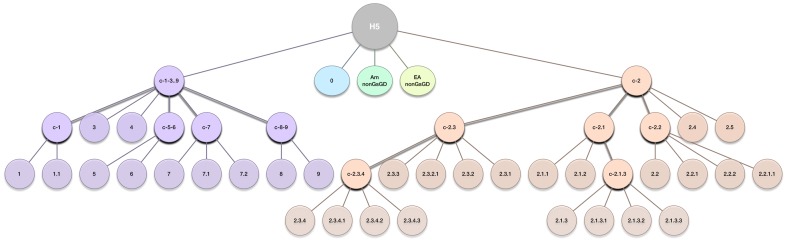
Annotation tree for LABEL's H5N1 annotation module. Each internal node corresponds to an annotation level (classification step) within the hierarchical annotation process. Accordingly, HMM profiles and SVM classes used by the H5 module are represented by all non-root nodes (color circles). The “c-*X*” notation stands for “cluster *X*,” where *X* is some general group of clades. Exact correspondence with the H5N1 clade clustering [5] is not preserved in the annotation tree for the sake of algorithmic simplicity.

**Figure 2:**
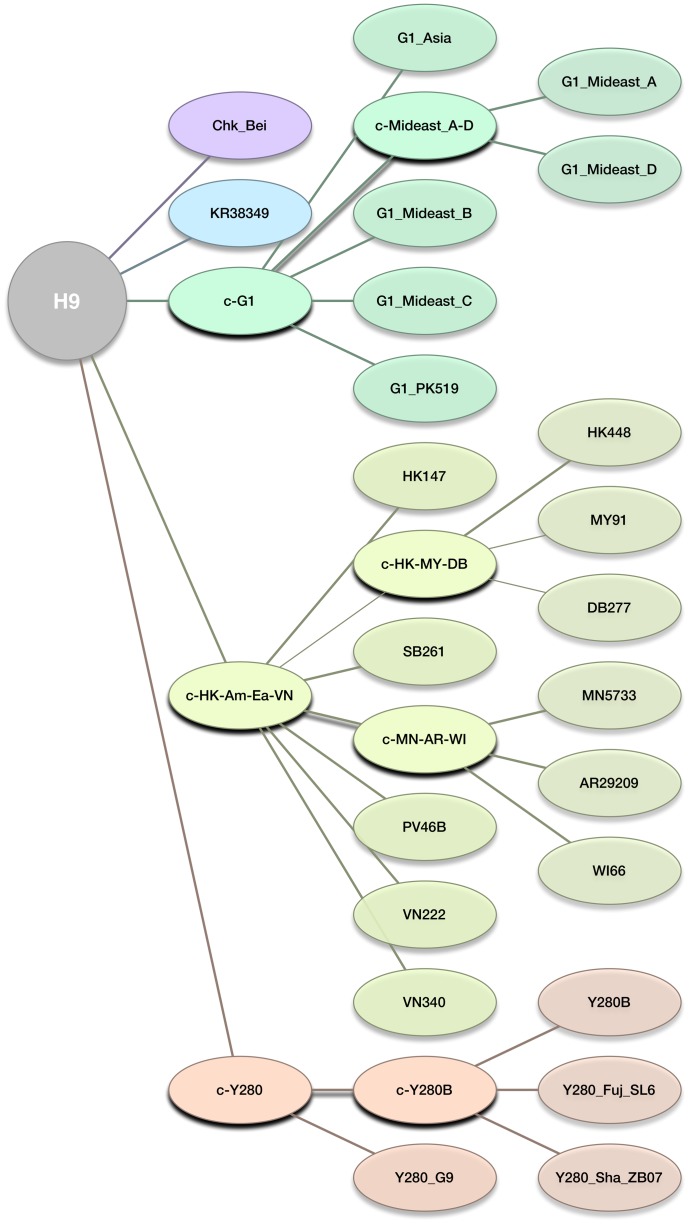
Annotation tree for LABEL's H9N2 annotation module. Each internal node corresponds to an annotation level (classification step) within the hierarchical annotation process. Accordingly, HMM profiles and SVM classes used by the H9 module are represented by all non-root nodes (color circles). The “c-*X*” notation stands for “cluster *X*,” where *X* is some general group of clades. Clades are named according to historical names and for representative sequences.

LABEL classifies gene sequences into evolutionary clades defined at the nucleotide level. We will refer to a set of clades defined on a phylogenetic tree to be its *lineage partition* (see Supplemental Fig. S1, step 1). A lineage partition may be used to develop an *annotation tree* resembling the partition's phylogeny and in turn create a hierarchy of pHMMs (step 2). Next one can train a hierarchy of SVMs (step 3) using smaller samples of the data and the pHMMs previously put in place. Each internal node in the annotation tree is connected to only a few annotation nodes which are known together as an *annotation level*. This hierarchical structure allows the annotation of gene sequences to proceed from a root level to the tips of the annotation tree with increasing specificity eventually being reached (step 4). As a consequence, lineages closer to the root are often combined with descendant clades and grouped as broader *clusters* of clades. Annotation trees for H5N1 and H9N2 are given in Figures 1 and 2, respectively. The depth and detail of the annotation tree need not be equal for every branch, and for some lineages only summary annotations may be desired (notice the short depth of the American [*Am_nonGsGD*] and Eurasian [*EA_nonGsGD*] non-Gs/GD lineages in Fig. 1).

Once a hierarchy of pHMMs has been established (Supplemental Fig. S1, steps 1 & 2), the rest of LABEL's SVM training, as well as lineage assignment, will follow two distinct phases: an *analysis phase* and a *decision phase* (steps 3 & 4). For the analysis phase, sequences are scored by pHMMs to analyze their similarity to defined clades or clusters within the annotation tree. In the decision phase, scoring data is used to train SVM decision-making as well as provide data for query sequence annotation. A completed LABEL module includes the full hierarchy of trained SVMs, pHMMs, and annotation labels.

### Training a LABEL module

LABEL was written as a shell pipeline using a BASH script calling many custom Perl scripts, standard UNIX utilities, and linking to various third-party binaries.

First, for the particular gene of interest, sequences from the same clade were divided into separate files. This was done using either existing annotations (H5N1) or through careful scrutiny of the phylogenetic tree information combined with historical considerations from the literature (H9N2). Each clade sequence library was aligned using MUSCLE v3.8.31 [29] with default parameters. JalView [30] was used to manually edit nucleotide alignment frame-shifts and trim the jagged ends of the alignment for greater consistency and quality. Specifically, the H5N1 hemagglutinin alignment was trimmed to GATCAGATT...GCACTGGCA with respect to A/goose/Guangdong/1/96 (AF144305/EPI_ISL_1254) while the H9N2 hemagglutinin alignment was trimmed to GATAAAATC...TCATCTCTT relative to A/chicken/Beijing/1/94 (AF156380/EPI_ISL_1270). Clade-specific alignments at each annotation level must not have mismatched alignment endings with respect to each other. Highly conserved motifs in the sparse ends can introduce unwanted bias towards a particular clade-specific HMM profile and degrade performance.

For LABEL's analysis phase, hidden Markov model profiles were constructed from clade nucleotide alignments using the modelfromalign program included in the SAM v3.5 package [23]. SAM's hmmscore program (with default settings) was used to score sequences with the clade-specific HMM profiles.

To train and make classifications for LABEL's decision phase, we chose a multi-class support vector machine implemented by the *Shogun Machine Learning Toolbox* [31] using the Generalized Minimal Norm Problem or GMNP [32] method and a non-homogeneous polynomial kernel of degree 20 (selected using leave-one-out cross-validation of preliminary data, results not shown) with data normalization. A binary SVM can be utilized when only 2 groups are present by calling LIBSVM [33] using *Shogun* under the same kernel parameters. SVM training data is composed of reverse-corrected log-likelihood scoring matrices with dimensions determined by the number of training sequences versus the number of clade-specific HMM profiles at the current annotation level. In other words, each training sequence is scored not only against its own clade-specific HMM profile but also against all other profiles within the current annotation level.

SVM training sequences were usually small representative samples from each clade or cluster. For very small clades, full sets were sometimes needed while for larger clades random sampling was the initial starting point (custom Perl script). To increase annotation accuracy, SVM training data for each clade was down or up-sampled in an *ad hoc* manner, especially if clade sizes were highly disproportionate. However, once a LABEL module was fully trained the annotation of unknown HA sequences required no phylogenetic expertise or intervention.

### Analysis of HA genes

After training a LABEL module for a desired influenza gene and lineage partition, clade annotation for any given set of sequences will proceed in a deterministic fashion. LABEL contains no user parameters or options that can affect the outcome of the lineage assignment operation, making the tool consistent and reliable. Similar to module training, sequences are scored by the HMM profiles (analysis phase) of the current annotation level in order to produce a matrix of scores for SVM classification and clade annotation (decision phase). Lineages are assigned hierarchically with the annotation becoming final once it reaches a leaf node in the annotation tree.

For each HA sequence analyzed, the output from LABEL includes a FASTA header and the corresponding predicted annotations in various file formats (plaintext, tab-delimited, etc.). Moreover, a trace of the pHMM scores is generated for each annotation level reached within the tree. FASTA sequence files corresponding to each annotated clade are also provided—the full query sequence set being available for optional re-annotation and alignment as well. Lastly, for manual verification of LABEL's annotations, one can optionally align and create a tree of query sequences combined with a small reference library stored within the LABEL module. In such a tree, predicted annotations of the query sequences will appear in the form of {*PRED:annotation*}. Maximum likelihood tree construction in LABEL is done using FastTree2 [34] with GTR+GAMMA and 1000 local support bootstraps. FastTree2 was also used in phylogenetic analyses throughout this study.

### Runtime estimation

LABEL was tested versus an Intel-optimized version of MUSCLE as well as MAFFT version 6.851b using FFT-NS-i [35]. The UNIX time utility was used for assessing runtime on a single 12-core 2.8 GHz Xeon computer with 48GB of RAM (LABEL takes advantage of multi-core architecture). Tests were run in quintuplicate with the number of seconds averaged for each sample size. Samples were taken randomly without replacement from the H5N1 dataset using a custom Perl script. A fixed set of 200 non-redundant reference sequences was added to each query set for MUSCLE and MAFFT to simulate the usual phylogenetic lineage inference process.

### Annotation accuracy

Accuracy, represented as a percentage, is the number of samples where LABEL correctly annotated the sequence divided by the total number of sequences to be annotated. Since more than two groups are annotated and because clades can be unequal in sample size, we chose the balanced error rate [36] or BER as a second, complementary measure of accuracy with 0% BER being best. BER is calculated by finding the percentage of incorrectly annotated samples for each group and averaging their error rates. The outlier groups (typically very small in size) are aggregated into single group for the purposes of the BER averaging (but not for the purpose of assessing incorrect annotations).

### Pairwise distance calculations

Pairwise distance matrices (*p*-distance) were calculated by MEGA5 [37] for use with between and within group averaging via custom Perl script, and by the R *ape* package [38] for use with classical multidimensional scaling in R [39], [40]. The *C-value* ratio used in the H9N2 lineage partitioning is the ratio of the average pairwise distance between a particular taxon and its closest neighboring group divided by the average pairwise distance within that selected clade. For distance matrices computed from pHMM scoring matrices, scores were first normalized [41] before taking the usual pairwise Euclidean distances.

## Results

### LABEL annotation of highly pathogenic avian influenza A(H5N1) viruses

Since the earliest detection of highly pathogenic H5N1 viruses, the immunologically critical viral surface protein, hemagglutinin, has diverged into 32 phylogenetically distinct clades with no fewer than 12 new genetically-defined clades emerging in just the past several years [5]. We have adopted the WHO/OIE/FAO H5N1 evolution working group's clade annotations for LABEL's H5 annotation module. Supplemental Figure S2 shows a representative guide tree of 581 H5N1 hemagglutinins with strain names annotated. Additionally, Figure 1 shows a diagram of the H5 annotation tree corresponding to all defined lineages.

As seen in Table 1, LABEL achieved 100% accuracy on self-validation data (HMM and SVM training data, see Supplemental Fig. S1) and, more importantly, on data excluding SVM training sequences (SVM test set). The minimum sequence length observed in these sets was 1,339 nts with an average full HA length around 1700 nts. The self-validation dataset contains many divergent sequences and outliers. Therefore, we have used it to assess the broadest range of clades in our sequence length analyses.

**Table 1:** Hemagglutinin clade annotation for avian influenza subtype A(H5N1) by LABEL.

Hemagglutinin clade annotation for avian flu H5N1	Sequence Count	Incorrect Annotations	Accuracy (BER)	Avg. Length (nts)	Min. Length (nts)
Gs/GD lineage H5N1 (SVM test set)	1920	0	100.0% (0%)	1707.3	1339
Plus SVM training set (self-validation set)	2506	0	100.0% (0%)	1707.5	1339
**full HA1 (GATCAGATT..AGAAATACC DQI..RNT)	2164	20	99.1% (1.9%)	959.5	919
HA1 frag. 1 (GATCAGATT..TTGAGCAGA DQI..LSR)	1161	137	88.2% (20.6%)	320.8	305
HA1 frag. 2 (ACAAACCAT..CCAGAAATA TNH..PEI)	1486	96	93.5% (10.3%)	317.8	315
**HA1 frag. 3 (GCTACTAGA..AGAAATACC ATR..RNT)	1228	157	87.2% (27.0%)	321.0	318
**HA1 fragments 1 + 3	1761	145	91.8% (27.5%)	641.7	612
*H5 submitted 2011/2/1 to 2012/4/1 (HMM & SVM test set)	373	0	100.0% (0%)	1706.3	1202
Non-GS/GD H5 (SVM test set)	465	0	100.0% (0%)	1722.0	1615

* Non-redundant H5N1, GISAID, Min. Len. 1200 bp, excluding previous sets, non-laboratory derived.

** The cleavage site is not included.

Start and stop 9-mers with translated amino acids are given for each fragment relative to A/goose/Guangdong/1/96 (AF144305/EPI_ISL_1254); accuracy is the number of correct clade annotations over the total number of tested HA nucleotide sequences; BER is the balanced error rate.

To evaluate whether partial H5 sequences from particular hemagglutinin regions can be used for accurate clade annotation, we tested LABEL lineage assignment on the HA1 segment as well as three nearly equal non-overlapping regions of HA1. We have observed that as many as 40% of smaller H5 HA sequences (less than 1200 nts) from public databases cover the HA1 region (95% of alignment length) while less than 2% of these partial HA cover the HA2 segment (data not shown). Table 1 shows that the use of HA1—on average about 960 nts—is highly informative, yielding over 99% accuracy with only 20 out of 2,164 non-redundant sequences being annotated erroneously (balanced error rate of 1.9%). This was expected as the HA1 segment is also generally more phylogenetically informative than the HA2 segment [42].

Performing LABEL annotation on non-overlapping fragments of HA1 (around 300 nts) reduced accuracy to between 87.2% (fragment 3) and 93.5% (fragment 2). Smaller H5 HA (less than 1200 nts) encompass regions corresponding to fragments 2 or 3 as much as 60% of the time while fragment 1 is represented 40% of the time (data not shown).

LABEL can properly interpret long deletions because it relies on pHMMs which in turn model potential deletion states. Thus, annotation accuracy was improved with the concatenation of fragments 1 and 3, from 88.2% and 87.2% when assessed individually to 91.8% when concatenated together. In brief, LABEL is capable of annotating viruses with good accuracy even on smaller fragments. However, annotation is generally more reliable with longer sequences.

To test LABEL accuracy and performance on data not used to develop our annotation modules in any way, we obtained 373 previously unidentified H5 sequences from GISAID submitted between 1 February 2011 and 1 April 2012. Of these, 119 were new with respect to date collected as well. Sequence annotations were compared to BLAST search and traditional manual phylogenetic annotation as described previously. For this new set LABEL achieved 100% clade annotation accuracy (see Supplemental File S1).

Finally, we assessed annotation performance for two very broad non-Gs/GD lineages: one for the Americas (*Am_nonGsGD*) and one for Eurasia (*EA_nonGsGD*). Such H5s may be mixed in with Gs/GD-like H5N1 hemagglutinins when querying public databases such that annotating them allows for proper discrimination and separation of H5 samples. Table 1 shows accurate annotation of all 465 non-Gs/GD lineage viruses tested. These results, combined with accuracy in annotating Gs/GD-like viruses (even partial HA1 sequences), demonstrate the applicability of LABEL for future H5N1 surveillance activities.

### LABEL annotation of low pathogenicity avian influenza A(H9N2) viruses

We have trained LABEL to do lineage assignment on H9N2 avian influenza viruses. Phylogeny of the H9 hemagglutinin has been reported in [43]–[46] and also in the aforementioned Feb. 2010 WHO report. However, in contrast to H5N1 viruses, it was necessary to first revise and annotate the H9 lineages. Our phylogenetic analysis, including a substantial number of recently deposited sequences that were not available for previous analyses (1,592 sequences in total), allowed for the classification of H9 HA genes into 23 distinct clades and 5 clade-specific outlier groups. Table 2 shows the average pairwise distances (APD) within each clade, the APD to the most closely related taxa, and the ratio (C-value) of these two APDs. Lineages were restricted to a within group APD of 6% and to C-values of no more than 1.1. Clade nomenclature was chosen based on previous classification as well as representative strain names within the clade of interest, as shown in Table 3. Figure 2 shows the annotation tree for LABEL's H9 annotation module (outlier groups are not shown for simplicity). Correspondingly, Supplemental Figure S3 shows a representative tree of 606 H9N2 hemagglutinins with strain names annotated.

**Table 2:** Revised 2012 H9N2 nomenclature.

Revised H9N2 clade name	Clade sample count	Closest clade	APD between clade & closest	APD within clade	C-value
AR29209	5	HK448	14.9%	2.7%	5.5
Chk_Bei	76	Y280_G9	7.0%	1.7%	4.1
DB277	7	HK448	8.5%	3.4%	2.5
G1_Asia	42	G1_PK519	4.5%	3.5%	1.3
G1_Mideast_A	39	G1_PK519	4.3%	2.3%	1.9
G1_Mideast_B	116	G1_PK519	6.0%	5.0%	1.2
G1_Mideast_C	9	G1_PK519	3.8%	1.9%	2.1
G1_Mideast_D	35	G1_PK519	3.7%	3.2%	1.2
G1_PK519	4	G1_Mideast_D	3.7%	1.3%	2.9
HK147	9	HK448	7.6%	3.5%	2.2
HK448	6	HK147	7.6%	5.4%	1.4
KR38349	55	PV46B	10.6%	4.8%	2.2
MN5733	28	HK147	9.2%	3.5%	2.6
MY91	3	HK448	10.8%	0.8%	13.3
PV46B	9	VN340	6.6%	3.9%	1.7
SB261	13	PV46B	8.0%	2.7%	2.9
VN222	3	PV46B	10.0%	4.1%	2.5
VN340	39	PV46B	6.6%	3.8%	1.7
WI66	6	MN5733	15.7%	0.9%	17.2
Y280_Fuj_SL6	54	Y280_G9	9.2%	1.3%	7.3
Y280_G9	171	Chk_Bei	7.0%	4.5%	1.5
Y280_Sha_ZB07	22	Y280_G9	7.6%	2.9%	2.6
Y280B	824	Y280_Sha_ZB07	7.8%	5.2%	1.5
TOTAL COUNT:	1592		Full tree APD:	10.0%	

Average pairwise distance (APD) values within and between closely related clades using a *p*-distance calculation with pairwise deletion. The C-value is the ratio of the between group APD to the within group APD.

**Table 3:** Revised 2012 H9N2 nomenclature references.

Representative Virus	Clade	Additional Info
A/quail/Arkansas/29209-1/93	AR29209	
A/shorebird/Delaware_Bay/277/2000	DB277	
A/duck/Hong_Kong/448/78	HK448	
A/duck/Malaysia/91/1997	MY91	
A/mallard/Ireland/PV46B/1993	PV46B	Supplemental Figure S3
A/shorebird/DE/261/2003	SB261	
*A/duck/Vietnam/NCVD-222/2009	VN222	
A/duck/Viet_Nam/340/2001	VN340	
A/Chicken/Korea/38349-p96323/96	KR38349	[54], [55]
A/duck/Hong_Kong/147/77	HK147	
A/goose/Minnesota/5733/80	MN5733	[44]
A/turkey/Wisconsin/1/1966	WI66	
A/quail/Hong_Kong/G1/97	G1_Asia	
A/chicken/Middle_East/ED-1/1999	G1_Mideast_A	
A/chicken/Iran/B102/2005	G1_Mideast_B	[43]
A/quail/Dubai/303/2000	G1_Mideast_C	
A/chicken/Saudi_Arabia/CP7/1998	G1_Mideast_D	
A/chicken/Pakistan/AG519/98	G1_PK519	aka Pakistan1999
A/chicken/Beijing/1/94	Chk_Bei	[56]
A/chicken/Hong_Kong/G9/97	Y280_G9	aka Y280A
A/duck/Hong_Kong/Y280/97	Y280B	[13]
A/chicken/Fujian/SL6/2011	Y280_Fuj_SL6	
A/chicken/Shandong/ZB/2007	Y280_Sha_ZB07	Supplemental Figure S3

* Named for virus not in our dataset that clusters with clade VN222.

Having trained LABEL on a dataset incorporating the revised H9 nomenclature, we tested accuracy as described for H5 above. Table 4 shows that LABEL was able to correctly identify clades for all of the full length HA genes analyzed. The test dataset was next subdivided into HA1 and HA2-encoding sequences as well as three ∼321 nt non-overlapping fragments encoding HA1—recapitulating the approach used to analyze the informativeness of partial regions within the H5 module. LABEL annotation accuracy was 99.9% and 96.8% when using non-redundant HA1 and HA2-encoding regions respectively. Interestingly, although fragments 1 and 2 of HA1 are relatively short, they retain very high annotation accuracies (>99%). By contrast, fragment 3 only provides 93.4% annotation accuracy, suggesting less informative or fewer variant sites reside in this region (Supplemental File S2). While our H9N2 lineage partition defines clades that are diverse in terms of their APD and sampling (Table 2), LABEL was still able to achieve 100% annotation accuracy on whole HAs and over 93% accuracy using just 321 nt length fragments (HA1).

**Table 4:** Hemagglutinin clade annotation for avian influenza subtype A(H9N2) by LABEL.

Hemagglutinin clade annotation for avian flu H9N2	Sequence Count	Incorrect Annotations	Accuracy (BER)	Avg. Length (bp)	Min. Length (bp)
H9N2 lineage HA(self-validation set)	1592	0	100.0% (0%)	1642.0	1202
Without SVN training data (SVM test set)	1250	0	100.0% (0%)	1640.9	1202
full HA1 (GATAAAATC..CCTGCTAGA DKI..PAR)	992	1	99.9% (0.7%)	959.9	924
HA1 fragment 1 (GATAAAATC..CTTTTTAGT DKI..LFS)	674	4	99.4% (3.2%)	318.0	315
HA1 fragment 2 (TCTGCTAGT..CCCCCTGTC SAS..PPV)	804	4	99.5% (2.5%)	321.0	320
HA1 fragment 3 (AATGGTCAG..CCTGCTAGA NGQ..PAR)	749	47	93.7% (5.2%)	312.0	311
full HA2 (AGAGGACTA..TCATCTCTT RGL..SSL)	843	27	96.8% (2.3%)	590.0	562

Start and stop 9-mers with translated amino acids are given for each fragment relative to *A/chicken/Beijing/1/94* (AF156380/EPI_ISL_1270); accuracy is the number of correct lineage annotations over the total number of tested HA nucleotide sequences; BER is the balanced error rate.

### Resolving clade annotations of transitional H5 HA genes with LABEL

Transitional HA sequences that bisect two sister clades in a phylogenetic tree pose a special challenge for annotation. For example, we have observed three H5N1 viruses that do not fit well with the older clade *1* or the newer clade *1.1*: A/duck/Vietnam/NCVD-126/2007, A/duck/Vietnam/NCVD-001/2008, and A/duck/Vietnam/NCVD-385/2009. We classify these viruses as *1.1-like* because they match most closely with *1.1* viruses using BLAST (data not shown). Other authors also note the separation of NCVD-126 from clade *1.1* [47]. Given the uncertainty in phylogenetic tree reconstruction, such problems in cladistic assignment and H5N1 annotation are surprisingly rare. Supplemental Figure S4 shows the tree for clades *1* and *1.1* along with the *1.1-like* group. The APD of the *1.1-like* group to clades *1* and *1.1* is 3.69% and 3.24% respectively, while its within group APD is 1.85%. From APD analysis, one may conclude that these viruses should not belong to either clade *1* or *1.1*, supporting their classification as “*1.1-like*”.

**Figure 3:**
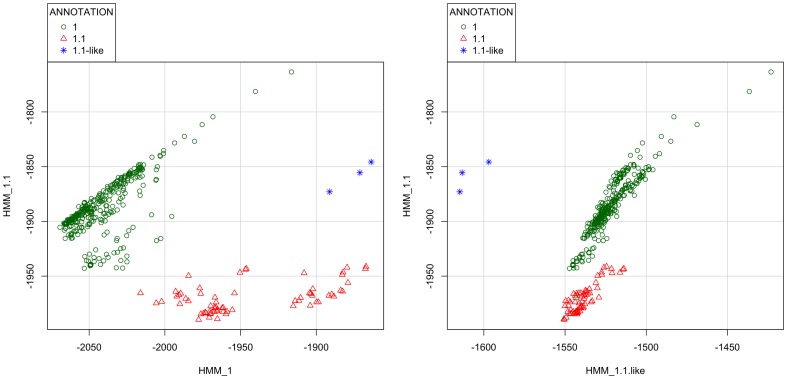
Two-dimensional scatterplots of profile HMM negative log-likelihood scores for H5N1 hemagglutinins in clades *1* (green circles) and *1.1* (red triangles) along with those in a *1.1-like* group (blue stars). (A) Plot shows scores for the clade *1.1*-specific pHMM (Y-axis) versus scores for the clade *1*-specific pHMM (X-axis). (B) As in A, but with the X-axis containing scores for the *1.1-like* pHMM instead. Smaller (more negative) numbers are considered better fits for that clade or group.

For corroboration of *1.1-like* clustering patterns, LABEL was used to generate scoring matrices for plotting using the R *car* package [*48*]. Figure 3A shows a scatterplot of HMM profile scores where the clade-*1*-specific profile scores are graphed versus clade*-1.1*-specific profile scores. This plot shows that *1.1-like* viruses do not cluster with either clade *1* or clade *1.1* hemagglutinins. Scoring the same sequences with an HMM profile created from the *1.1-like* group and plotting it versus the clade *1.1* pHMM shows a distinct separation of *1.1-like* sequences from the other clades (Fig. 3B).

### Clade annotation of “outlier” H5 HA genes with LABEL

Clade *3* viruses were identified as early as 2000 while the related clade *4* appeared in two waves—2002 to 2003 and 2005 to 2006. According to WHO/FAO/OIE's 2011 nomenclature guide tree (www.who.int/influenza/gisrs_laboratory/201101_h5fulltree.pdf) three viruses were identified as “outliers” clustering near clades *3* and *4*: A/chicken/Shantou/904/2001, A/Hong_Kong/378.1/2001, and A/duck/Hong_Kong/380.5/2001. We will refer to this outlier group as *3-like*. The determination of such outliers may be related to the nomenclature criteria (such as violating APD thresholds) or by clustering between established clades. Importantly, such determinations are very data-dependent and older annotations may cease to be appropriate as new samples are obtained.

**Figure 4:**
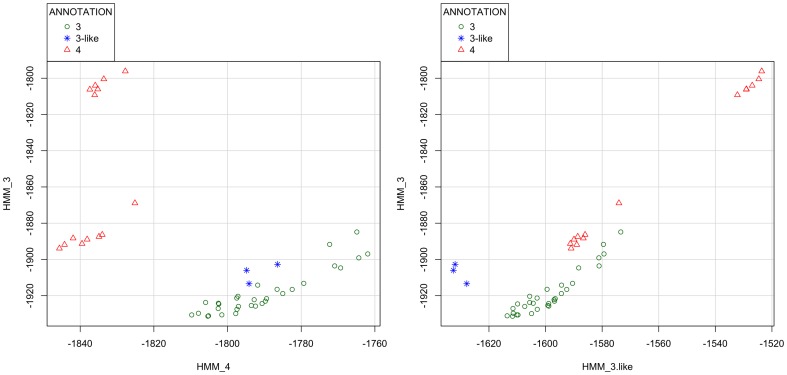
Two-dimensional scatterplots of profile HMM negative log-likelihood scores for H5N1 hemagglutinins in clades *3* (green circles) and *4* (red triangles) along with those in a clade *3-like* group (blue stars). (A) Plot shows scores for the clade *3*-specific pHMM (Y-axis) versus scores for the clade *4*-specific pHMM (X-axis). (B) As in A, but with the X-axis containing scores for the *3-like* pHMM instead. Smaller (more negative) numbers are considered better fits for that clade or group.

Phylogenetic tree and LABEL scatterplot analyses showed that these “outliers” were more similar to clade *3* than to clade *4*. The tree in Supplemental Figure S5 confirms that the *3-like* group clusters within clade *3*. The *3-like* group has an APD of 0.5% within itself and an APD of 2.47% between it and clade *4*. By contrast, the between group APD of the *3-like* group versus clade 3 is only 1.32%, violating the WHO/OIE/FAO requirement that clades have at least 1.5% APD between each other [17]. Moreover, the within group APD of clade *3* acceptably goes to 1.22% when the *3-like* sequences are added to it (within group APDs must be no more than 1.5%). Correspondingly, the pHMM scores of the *3-like* group cluster within clade *3* (Fig. 4A) although one can induce modest separation using a *3-like* group-specific HMM profile (Fig. 4B). The scoring data obtained through LABEL suggest that the *3-like* viruses could have been added to clade *3*, in agreement with phylogenetic clustering and distance evidence. Nonetheless, we retain the *3-like* nomenclature to be more consistent with WHO/FAO/OIE H5N1 evolution working group's latest recommendations and the purported disappearance of the H5N1 HA clade in recent years [5].

### Runtime performance

LABEL differs from tree-based clade annotation methods in that it does not require the alignment of query sequences with a reference library or the construction of a bootstrapped phylogenetic tree. Moreover, neither inferring lineages from the tree programmatically nor manually inspecting the tree is required to assign lineages. In our experience, the alignment phase of this process is the most time-consuming. Hence, it may be used as a baseline time for any tree-based method. For comparison of query sequence annotation runtimes of tree-based methods versus LABEL, we used an Intel-optimized MUSCLE binary and MAFFT to perform alignments on H5N1 hemagglutinins of Gs/GD-like viruses (a typical use case scenario).

For each query sample size (16, 32, 64, 128, 256, 512, and 1024 sequences) and adding in a constant 200 sequences for the alignment programs, we ran LABEL, MAFFT, and MUSCLE with 5 replications each. The guide tree available for H5 hemagglutinins on WHO's website consists of 196 sequences; therefore, 200 reference sequences is a realistic estimated sample size. Supplemental Figure S6 shows the averaged runtimes for LABEL, MAFFT, and MUSCLE. Our first observation was that the run time for LABEL is directly proportional to the size of the data set, while the run time for MUSCLE and MAFFT is proportional to the square of the size of the data set. LABEL and MAFFT have a similar run time while MUSCLE is slower than both. Alignment run time will also increase or decrease with respect to the reference dataset size. Finally, our benchmark compares the full LABEL annotation procedure to just the alignment stage of tree-based annotation pipelines, for which phylogenetic tree construction and lineage inference will also need to be performed. Full data for Supplemental Figure S6 is given in Supplemental File S3 along with additional runtimes for LABEL with MUSCLE-based control alignment features turned on.

### Dealing with inappropriate data using pHMM scores

LABEL's “best fit” clade annotation scheme can allow for the classification of inappropriate data (negative samples) as false positives. For example, the user might accidentally submit the wrong HA subtype, an amino acid sequence (rather than a nucleotide one), or the wrong gene segment. To mitigate such error, we established data filtering by profile hidden Markov model scores using module-defined thresholds as described in Supplemental File S4/Supplemental Methods. Such thresholds allow for an expected worst-case (350 nt samples) misclassification rate of less than 0.1% while retaining sensitivity to all positive samples (sequences for which the module has defined lineages).

Our filtering method demonstrates that sequence annotation and classification using pHMM scores alone can be highly effective. However, pHMM-only classification will be the most accurate for well-separated datasets, such as the H5 vs. H1-H4, H6-H16 hemagglutinin dataset shown in Supplemental File S4. For a more complex and highly similar set of clades, the pHMM/SVM strategy employed by LABEL can increase accuracy. To show this, we used pHMM-only clade annotation (best score wins) to re-classify the 2506 H5 HA shown in Table 1 and 1592 H9 HA shown in Table 4 over the same annotation trees and profile HMMs. Compared to LABEL's 100% accuracy on the H5 and H9 datasets, pHMM-only clade annotation were 81.7% (55.8% BER) and 97.5% accurate (17.9% BER) respectively.

## Discussion

### Hierarchical clade annotation

LABEL employs a hierarchical approach to lineage assignment that offers several benefits. First, as new viral clades emerge, adding a new leaf node to the module's annotation tree may not require the alteration of training data for older parent clades. Second, annotation of sequences using a nested set of clades eliminates unnecessary analysis of unrelated lineages. Since influenza genes very rarely undergo recombination this is appropriate. Finally, using a hierarchy allows one to discriminate between very similar sequences by narrowing down the scope of comparisons.

LABEL's hierarchical lineage assignment method resembles so-called “rank-flexible” techniques used in metagenomics [28], [49]. LABEL differs in that its hierarchical branching always continues until a leaf node is reached within the annotation tree, in that annotations may be defined at arbitrary levels of specificity not corresponding to traditional taxonomic units and, more importantly, that LABEL can be used for higher resolution typing within a species. As such, LABEL is well suited for the annotation of rapidly evolving RNA viruses.

In particular, heterogeneous clade specificity within LABEL's annotation tree allows researchers the flexibility to define lineage partitions *ad hoc*, as exemplified by the H5 module where further clarification of the *EA_nonGsGD* and *Am_nonGsGD* lineages was not defined or desired. Shallow clade annotation tree depth could also result from the clade going extinct or from a lack of clade samples.

Clade definition flexibility was important in creating our refined H9N2 nomenclature (Tables 2-3; Fig. 2; Supplemental Fig. S3) where sample size sometimes forced narrowly-defined lineages (*MY91*, *VN222*, *etc.*) or allowed more broad definition for others (*e.g., KR38349* and *Y280B*, within group APD of 4.8% and 5.2% respectively). Indeed, the choice of a broad within group APD threshold (6%) lets our lineage partition accommodate older, under-sampled, and possibly extinct groupings while our use of a minimum *C-value* (1.1) ensures clades will be less diverse internally than they are versus the nearest clade.

By contrast, increasing H5N1 surveillance allows the WHO/OIE/FAO H5N1 evolution working group nomenclature to limit within group APDs to <1.5% and between group APD to >1.5% for its unified nomenclature [5], [16]–[18]. To compare with our H9 nomenclature, the H5 unified nomenclature would use minimum *C-value* of 1—albeit with stricter thresholds. In addition, WHO/FAO/OIE specifies clades must contain at least four samples, be monophyletic, and have a minimum of 60% bootstrap support for Gs/GD-like viruses. LABEL does not rely on *explicitly* evaluating these unified nomenclature criteria as a phylogenetic tree method would; rather, LABEL does so *implicitly* by assigning a “best fit” clade consistent with WHO/OIE/FAO's lineage partition.

### Annotation of partial and transitional HA

LABEL annotated full length hemagglutinins correctly (100% accuracy) for both H5N1 and H9N2. However, due to technical difficulties, full-length sequences are not always available in surveillance. The use of partial sequences showed 87% to 99% accuracy depending on factors such as sequence length and the choice of region. The examined partial H5N1 hemagglutinin regions could not be annotated as well as those of H9N2 HAs (87.2%–99.1% vs. 93.7%–99.9% respectively). While the complexity of H5 lineages is greater than for H9 (32 clades in H5 versus 20 in H9; 7 outlier groups vs. 5 outlier groups), this difference in accuracy for the HA1 region sequences (a common partial sequence) may be traced to so-called outlier groups. Supplemental File S1 shows that of the 20 incorrectly annotated H5 HA1 sequences, 13 belonged to outlier groups with the majority being re-assigned to closely related clades. (We have refined outlier annotations to include clade context such as *2-like*, *1-8-like* [*1* or *8*-like], and *2.3.4-like* groups, see Supplemental Figure S2.) For the H5 HA1 segment dataset we chose to include the full dataset encompassing many outliers while for H9 HA1 dataset we excluded classifier training data and with it many outliers. It makes intuitive sense that transitional viruses will be more difficult to annotate from partial sequences, as particular gene regions may contribute to clade uniqueness.

Anomalous sequences within well-defined clades can also cause problems. For the H5 HA1 dataset, only A/pigeon/Laos/NCVD-36/2007 was classified to a distant clade (*1* instead of *2.3.4*). We have observed that adding NCVD-36 to a tree of full length HA introduces a topological inconsistency where the *1.1-like* group of outliers clusters instead with clade *2.3.4*. Using BLAST as well as phylogenetic analysis after removing NCVD-36 confirms that the *1.1-like* group should remain basal to clade *1.1*. Since LABEL correctly classifies NCVD-36 as *2.3.4* when the full-length sequence is provided, we believe that some discriminating information must exist in HA2 necessary for proper sequence classification. Using smaller sequence fragments for annotating H9 and H5 may sometimes be less reliable.

### Critical evaluation of LABEL

LABEL provides a method for fast (linear time, <1 second per sequence on H5) and highly accurate lineage assignment. Profile HMM scores collated in the LABEL pipeline can also be used to create both two and three-dimensional scatterplots, and we have shown a couple cases where scatterplot clustering can be used in conjunction with phylogenetic tree clustering to resolve difficult to annotate sequences.

In terms of runtime, BLAST-based annotation methods, such as [19], should be comparable or slightly faster than LABEL although BLAST runtimes will also be dependent on the size of the reference library. Decreasing the size of the reference library to improve runtime for BLAST may increase the chance of improperly annotating query sequences. Moreover, the choice of a fixed percent identity and e-value threshold for BLAST may fail to produce accurate matching over time as newer sequence data diverges from the reference library. BLAST-based lineage assignment methods will therefore be relatively fast but at the cost of accuracy (especially over time). In contrast, the usual tree-based lineage assignment method will be relatively accurate, but also relatively slow.

How then does LABEL handle continuing clade evolution? In our experience with training LABEL, we have noticed that sequences near the tips of a clade within a phylogenetic tree are reliably annotated with respect to different training sets whereas sequences nearer to the split between clades were not as reliably annotated. In order to empirically understand the effects of future sequence divergence on LABEL, we annotated H5 clades *1.1*, *2.2.1*, and *2.3.2.1* from our new H5 dataset (collected February 2011 to April 2012, not used in any training) with a “new” suffix to distinguish new sequences from older ones of the same name. Then, in Supplemental Figure S7, we created scatterplots for each cluster of clades by taking 2D classical multidimensional scaling of p-distance matrices vs. the scaling of distance matrices derived from the pHMM scores used by LABEL (scripts available upon request). New (blue) points unsurprisingly show drift from old ones (red) when visualizing p-distance (panels A1, B1, and C1 for clades *1.1*, *2.2.1*, and *2.3.2.1* respectively). On the other hand, clustering between old and new data is better preserved for plots computed from pHMM scoring matrices (A2, B2, and C2). It must be noted that defining new sub-clades, instead of relying on old definitions, is the work of subject matter experts and may take into account antigenic as well as genetic information. As such, LABEL was written to annotate existing nomenclatures and not invent new ones (although visualization from scoring matrices may help in this process).

In recent years, another methodology called phylogenetic placement has been of interest for short read placement on a reference phylogenetic tree [50], [51]. By contrast, LABEL is a hierarchical classifier complementary to such methods but very different in function. After the writing of this manuscript, the Influenza Research Database (IRD) [52] implemented an H5N1 clade annotation tool using *pplacer* as one of its components. We tested IRD's tool using full-length non-outlier H5 HAs for Goose/Guangdong-lineage viruses (2463 sequences). IRD's tool performed at 98.9% accuracy, while, anecdotally, the computation required over 3 times as much time compared to a local 3.2 GHz Quad-core Intel Xeon workstation (data not shown). A direct comparison may be performed if the work is published. However, tools like IRD's may be seen as *complementary* to LABEL and we hypothesize that several different methods in conjunction might produce conservative but reliable results. On the other hand, if reference dataset privacy concerns are an issue for the stand-alone distribution of software, LABEL has an advantage over alignment-based methods (traditional approach, BLAST or phylogenetic placement) in that module distribution does not require the distribution of reference sequences, even though reference sets may be optionally provided.

LABEL has several limitations that will be addressed in future versions. First, training LABEL modules requires both expertise in phylogenetics and an intimate knowledge of our pipeline. We plan to provide tools to streamline module training. Second, LABEL is very specific when it comes to detecting previously characterized “outlier” sequences but somewhat inflexible at detecting new ones. We plan to implement anomaly detection helpful for outliers and previously un-sampled lineages. Finally, while support vector machines are considered robust methods for machine-learning [53] and while HMM profiles provide a statistical framework for evaluating similarity to clade alignments, LABEL gives no indication of statistical confidence for its annotations. Methods for *p-value* estimation and other confidence measures are being investigated for the next major version of LABEL.

### Final remarks

We introduce a method for the rapid HA clade annotation of highly pathogenic H5N1 and low pathogenicity H9N2 influenza A viruses, two viruses with pandemic potential. However, we believe that this methodology may be developed to rapidly assess the full influenza genome in order to detect viral reassortment and identify novel influenza genotypes for epidemiological analyses. Moreover, while influenza was a useful organism to study due to its rapid mutation rate and the wealth of surveillance data available, we are not limited to influenza. The LABEL methodology outlined in Supplemental Figure S1 could be applied to any gene of interest provided one is able to develop a lineage partition for the various taxa involved, and provided the sequences are independently heritable. We believe that the rapid and accurate annotation of clades for human pathogens will aid molecular epidemiologic assessment and support public health interventions.

### Availability

We offer a web platform for LABEL along with its code and current annotation modules (H5 and H9) for use and download at *label.phiresearchlab.org*. We include instructions for easy installation on both Mac OS X and Linux systems. All of the custom LABEL scripts are licensed under the GNU Public License (GPL) version 3. Third-party licenses for some of LABEL's components (including GPL and a not-for-profit academic or government license for SAM) are included in the package and apply when used within LABEL or separately. For those wishing to host LABEL on their own webserver we have implemented options for cluster integration compatible with Open Grid Scheduler/Grid Engine 2011.11 (not used in benchmarks).

## Supporting Information

Figure S1 Diagram of LABEL module training and clade annotation.
(PDF)

Figure S2 Phylogenetic tree of H5 HA with annotated clades and strain names.
(PDF)

Figure S3 Phylogenetic tree of H9 HA with annotated clades and strain names.
(PDF)

Figure S4 Phylogenetic tree showing H5 outlier group *1.1-like*.
(PDF)

Figure S5 Phylogenetic tree showing H5 outlier group *3-like*.
(PDF)

Figure S6 LABEL benchmark versus alignment with 200 guide sequences.
(PDF)

Figure S7 Clustering of new H5s relative to old clades, pHMM scores vs. p-distance.
(PDF)

File S1 H5 dataset information & GISAID acknowledgement.
(XLSX)

File S2 H9 dataset information & GISAID acknowledgement.
(XLSX)

File S3 Data tables for LABEL benchmarks.
(XLSX)

File S4 Supplemental Methods on inappropriate data filtering.
(PDF)
