# Supplementary material for: LABEL: Fast and Accurate Lineage Assignment with Assessment of H5N1 and H9N2 Influenza A Hemagglutinins
Source: PLoS One. 2014 Jan 23;9(1):e86921. doi: 10.1371/journal.pone.0086921 (PMC3900692; doi:10.1371/journal.pone.0086921)
Supplement: Figure S1 — Diagram of LABEL module training and clade annotation. (PDF) [file pone.0086921.s001.pdf]

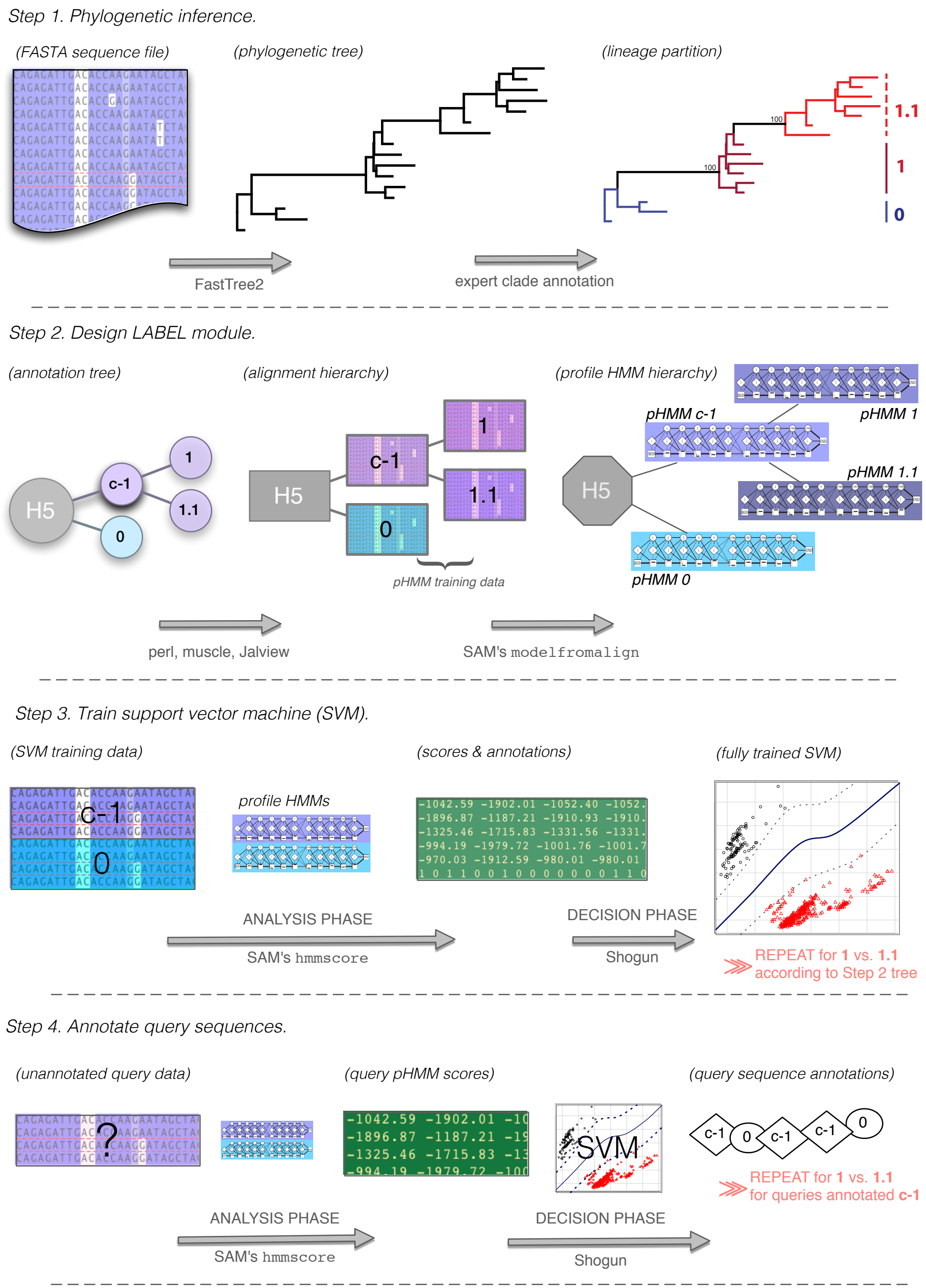

Supp. Fig. 1: The lineage partition in step 1 is used to design the annotation tree in step 2, giving rise to a hierarchy of profile HMMs. Step 3 is repeated at each internal node of the annotation tree, where profile scores of training data are used to train SVMs. For annotating query sequences, step 4 uses the pHMMs and SVMs from steps 2 & 3 to assign query sequence annotations in a root-to-tips fashion corresponding with the annotation tree.
