# Supplementary material for: LABEL: Fast and Accurate Lineage Assignment with Assessment of H5N1 and H9N2 Influenza A Hemagglutinins
Source: PLoS One. 2014 Jan 23;9(1):e86921. doi: 10.1371/journal.pone.0086921 (PMC3900692; doi:10.1371/journal.pone.0086921)
Supplement: Figure S2 — Phylogenetic tree of H5 HA with annotated clades and strain names. (PDF) [file pone.0086921.s002.pdf]

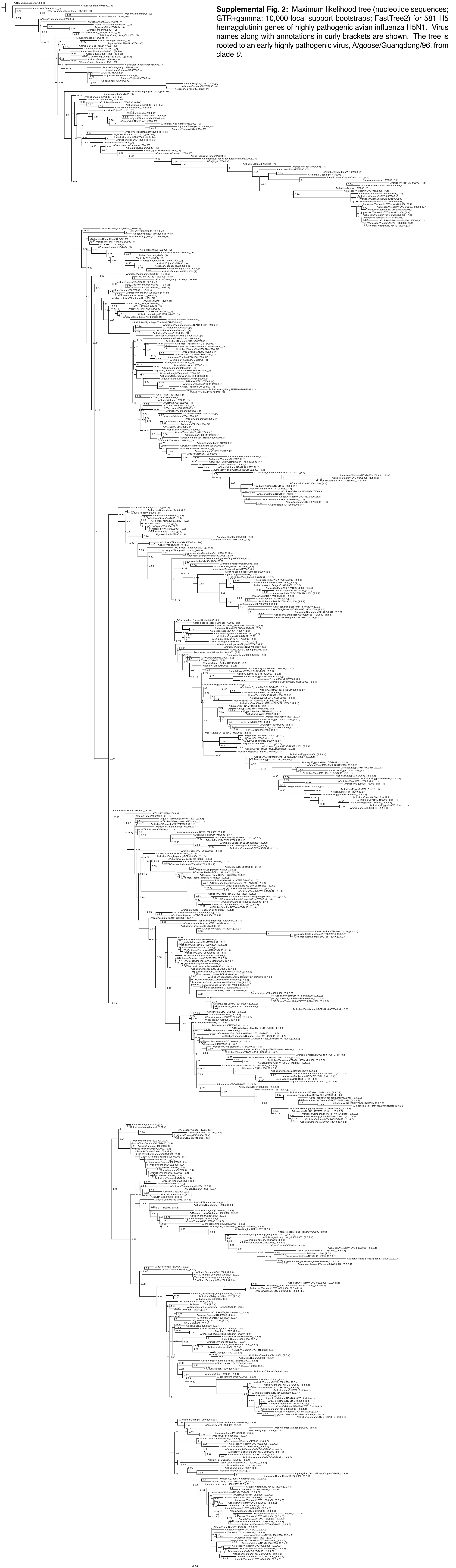

Supplemental Fig. 2: Maximum likelihood tree (nucleotide sequences; GTR+gamma; 10,000 local support bootstraps; FastTree2) for 581 H5 hemagglutinin genes of highly pathogenic avian influenza H5N1. Virus names along with annotations in curly brackets are shown. The tree is rooted to an early highly pathogenic virus, A/goose/Guangdong/96, from clade *0*.
