# Supplementary figures and images for: LABEL: Fast and Accurate Lineage Assignment with Assessment of H5N1 and H9N2 Influenza A Hemagglutinins
Source: PLoS One. 2014 Jan 23;9(1):e86921. doi: 10.1371/journal.pone.0086921 (PMC3900692; doi:10.1371/journal.pone.0086921)

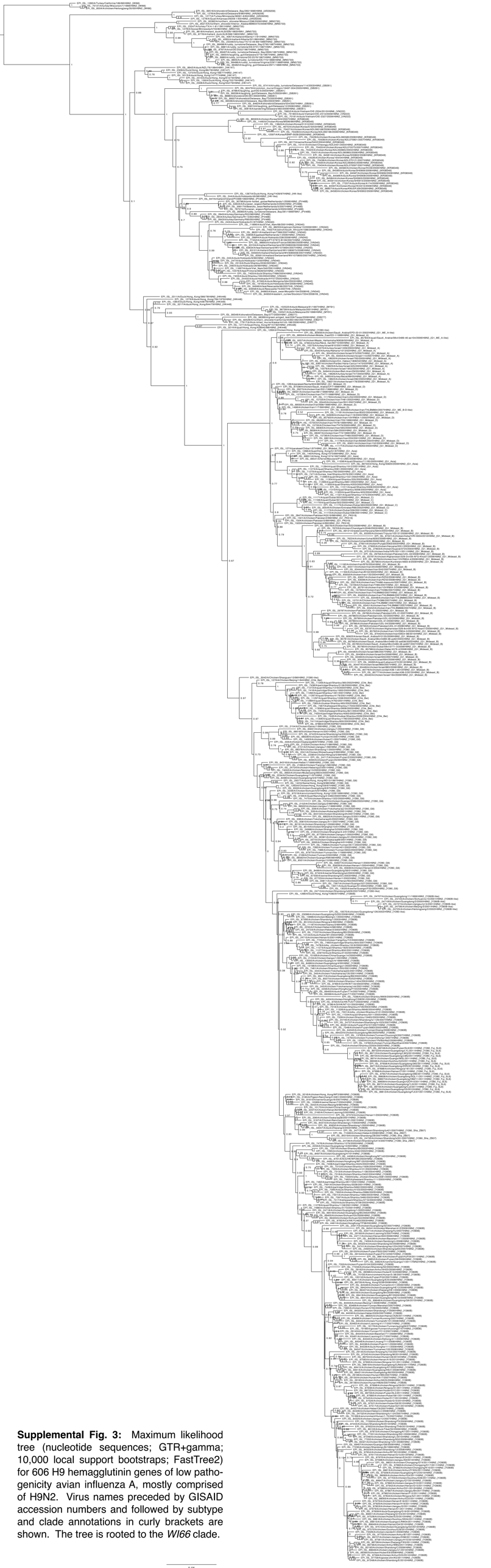

Supplement: Figure S3 — Phylogenetic tree of H9 HA with annotated clades and strain names. (PDF) [file pone.0086921.s003.pdf]
