# Supplementary material for: LABEL: Fast and Accurate Lineage Assignment with Assessment of H5N1 and H9N2 Influenza A Hemagglutinins
Source: PLoS One. 2014 Jan 23;9(1):e86921. doi: 10.1371/journal.pone.0086921 (PMC3900692; doi:10.1371/journal.pone.0086921)
Supplement: Figure S5 — Phylogenetic tree showing H5 outlier group 3-like. (PDF) [file pone.0086921.s005.pdf]

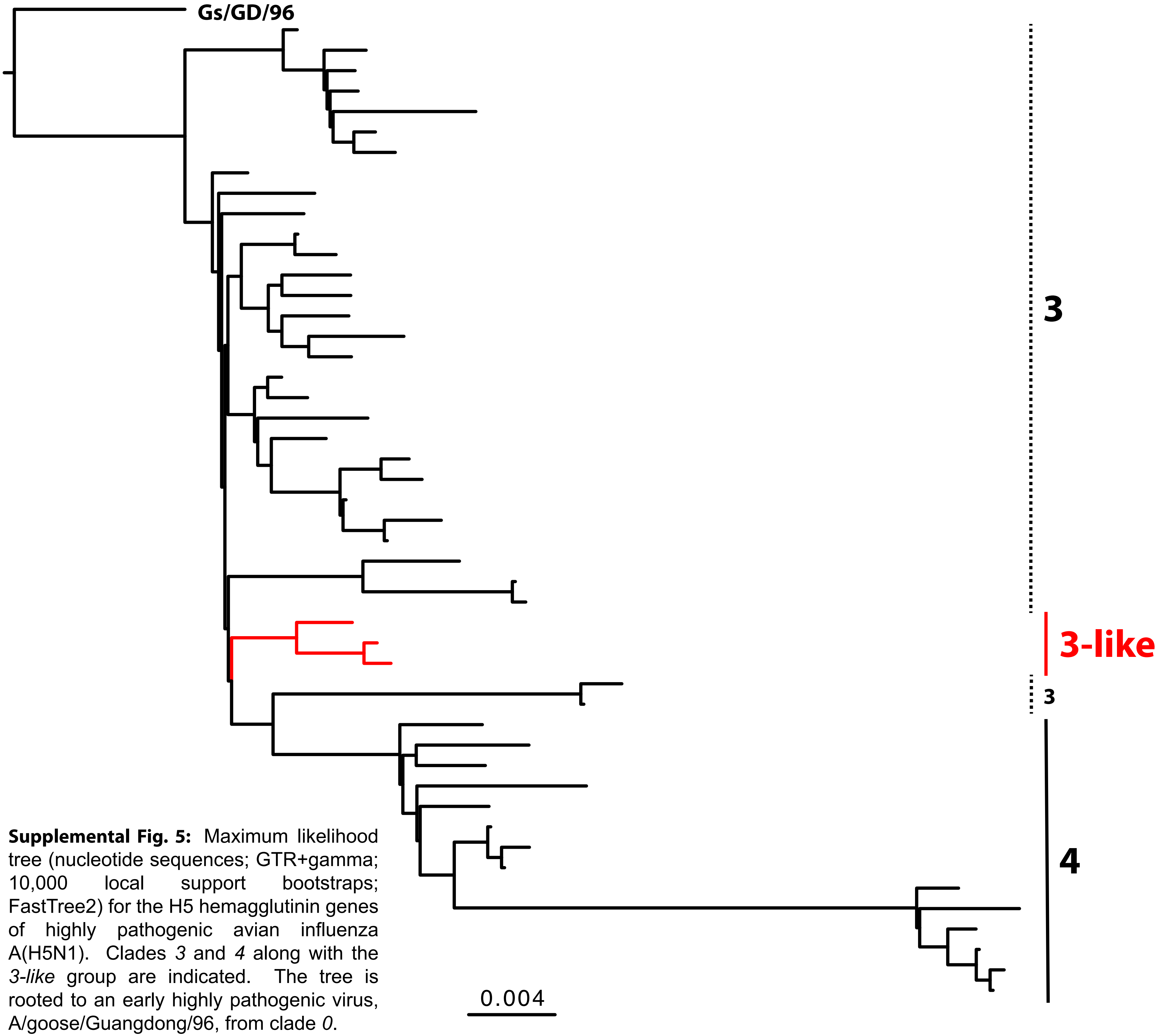


**Supplemental Fig. 5:** Maximum likelihood tree (nucleotide sequences; GTR+gamma; 10,000 local support bootstraps; FastTree2) for the H5 hemagglutinin genes of highly pathogenic avian influenza A(H5N1). Clades *3* and *4* along with the *3-like* group are indicated. The tree is rooted to an early highly pathogenic virus, A/goose/Guangdong/96, from clade *0*.
