# Supplementary material for: LABEL: Fast and Accurate Lineage Assignment with Assessment of H5N1 and H9N2 Influenza A Hemagglutinins
Source: PLoS One. 2014 Jan 23;9(1):e86921. doi: 10.1371/journal.pone.0086921 (PMC3900692; doi:10.1371/journal.pone.0086921)
Supplement: Figure S6 — LABEL benchmark versus alignment with 200 guide sequences. (PDF) [file pone.0086921.s006.pdf]

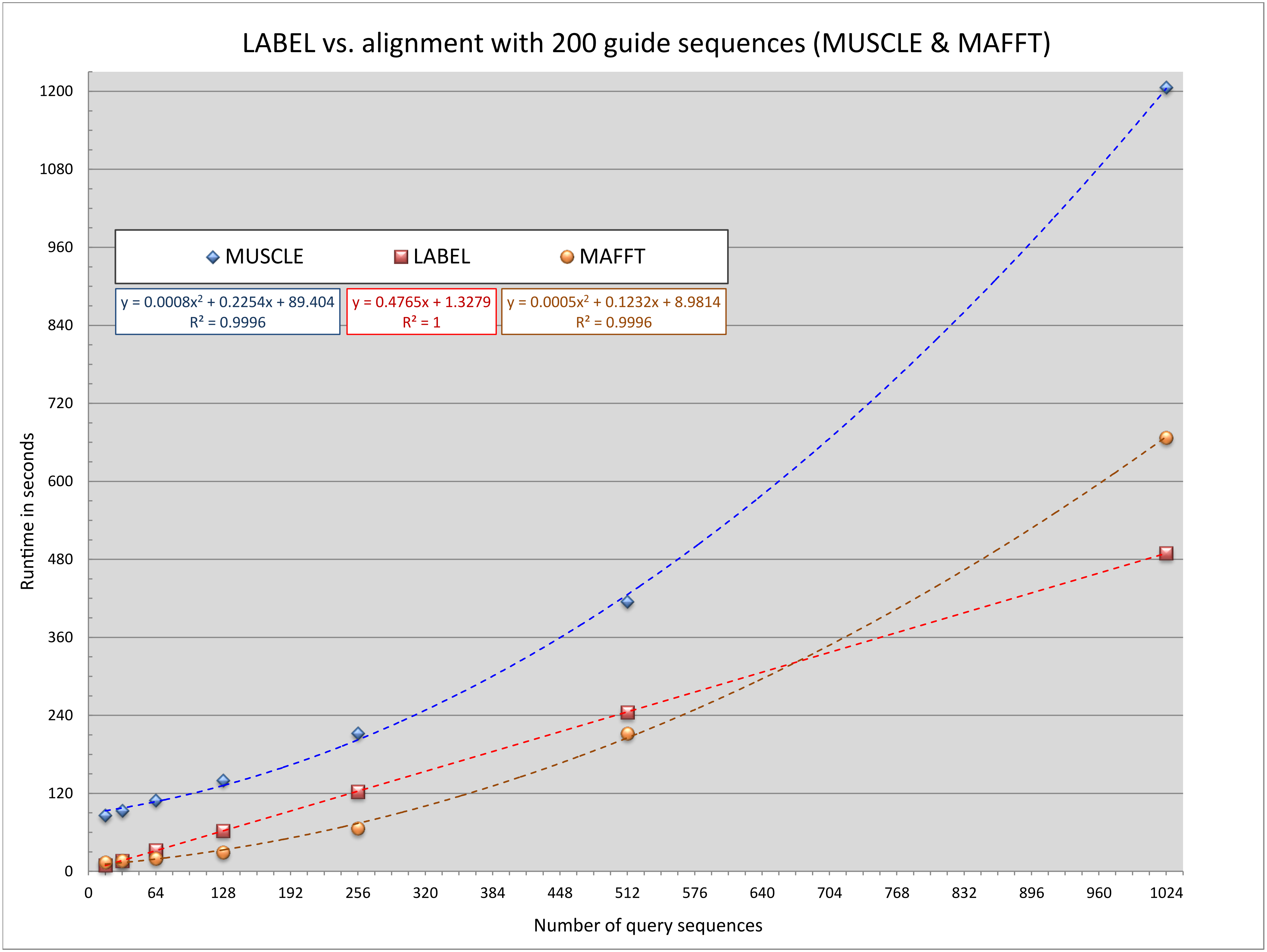

Supplemental Fig. 6: Number of seconds per query sequence for LABEL (red square), MAFFT (orange circle), and MUSCLE (blue diamond) to execute on a single 12-core 2.8 Ghz Xeon computer. The query dataset is composed of hemagglutinin gene sequences from highly pathogenic influenza A(H5N1) viruses. For MUSCLE and MAFFT, an additional 200 sequences are aligned with query sequences to simulate using a guide tree for annotation purposes. LABEL runtime is fitted linearly while MAFFT and MUSCLE are fitted to quadratic polynomials; the R-squared and equation of the line are shown.

Supplemental Fig. 6: Number of seconds per query sequence for LABEL (red square), MAFFT (orange circle), and MUSCLE (blue diamond) to execute on a single 12-core 2.8 Ghz Xeon computer. The query dataset is composed of hemagglutinin gene sequences from highly pathogenic influenza A(H5N1) viruses. For MUSCLE and MAFFT, an additional 200 sequences are aligned with query sequences to simulate using a guide tree for annotation purposes. LABEL runtime is fitted linearly while MAFFT and MUSCLE are fitted to quadratic polynomials; the R-squared and equation of the line are shown.
