# Supplementary material for: LABEL: Fast and Accurate Lineage Assignment with Assessment of H5N1 and H9N2 Influenza A Hemagglutinins
Source: PLoS One. 2014 Jan 23;9(1):e86921. doi: 10.1371/journal.pone.0086921 (PMC3900692; doi:10.1371/journal.pone.0086921)
Supplement: Figure S7 — Clustering of new H5s relative to old clades, pHMM scores vs. p-distance. (PDF) [file pone.0086921.s007.pdf]

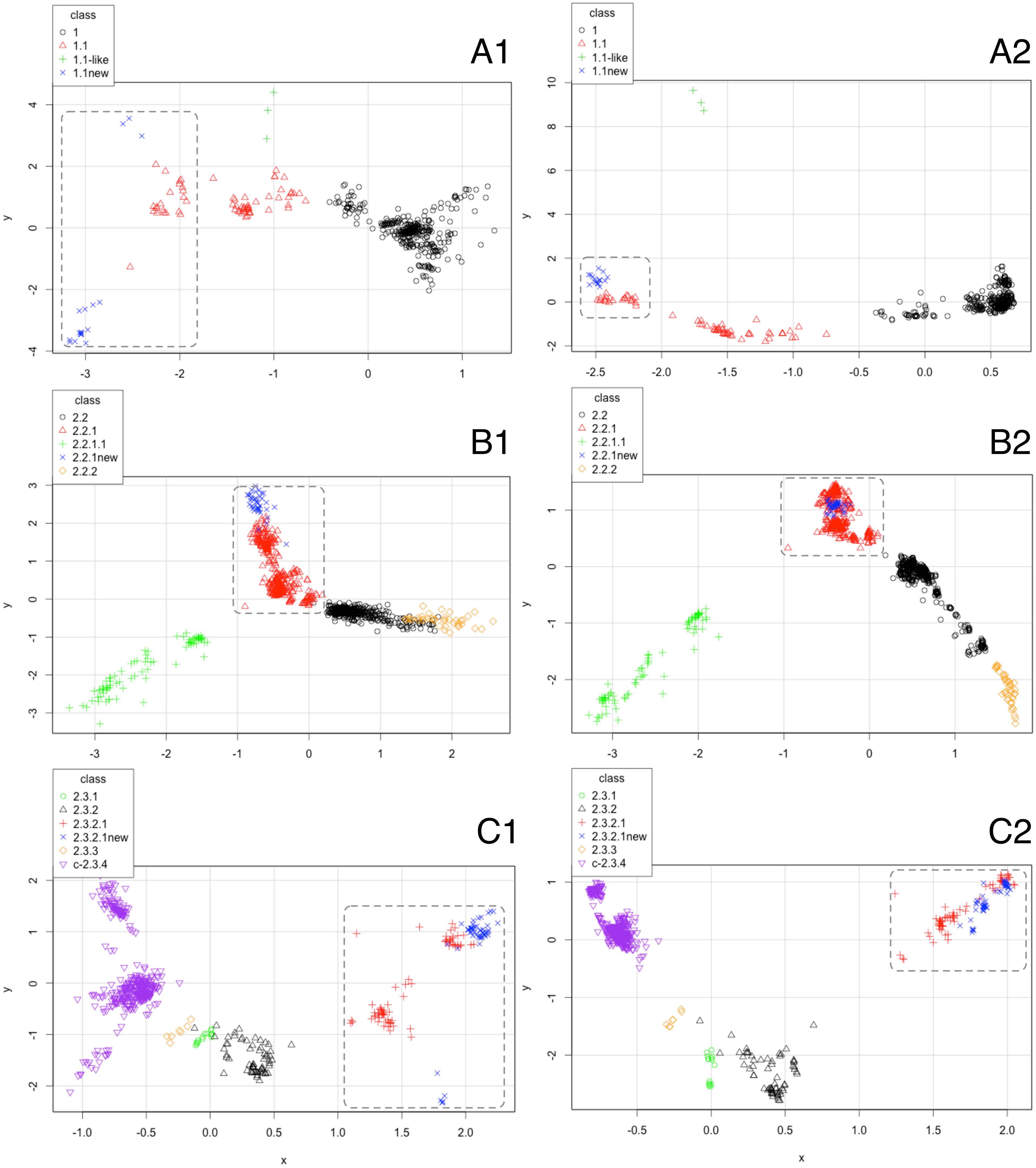

Supplemental Fig. 7: H5N1 hemagglutinin clade visualization showing the clustering of new data collected February 2011 to April 2012. Scatterplots of p-distance matrices from nucleotide sequence alignments are compared to distance matrices of normalized pHMM scoring matrices using 2D multidimensional scaling with standardized axes. Plots based on p-distance are (A1), (B1), and (C1) while plots (A2), (B2), and (C2) are based on pHMMs. For each plot, red points show the clade of interest while blue points show new data from the same clade. New data was not used for pHMM training; pHMM scores were generated for each old grouping. Clustering of red and blue points indicate similarity between old and new clade samples according to each method.
