# Supplementary material for: LABEL: Fast and Accurate Lineage Assignment with Assessment of H5N1 and H9N2 Influenza A Hemagglutinins
Source: PLoS One. 2014 Jan 23;9(1):e86921. doi: 10.1371/journal.pone.0086921 (PMC3900692; doi:10.1371/journal.pone.0086921)
Supplement: File S4 — Supplemental Methods on inappropriate data filtering. (PDF) [file pone.0086921.s011.pdf]

Supplemental File S4/Supplemental Methods

Inappropriate data filtering

LABEL classifies lineages for specified proteins and/or protein subtypes. However, there is nothing to prevent the user from submitting inappropriate data such as amino acid sequence (rather than nucleotide sequence), the wrong protein, or the wrong subtype. We have observed that databases such as GenBank may occasionally contain mislabeled influenza HA/NA subtypes. In order to prevent false classification of inappropriate data we have provided a filtering mechanism (optional to the creator of the module) and give guidelines for establishing a statistically derived filtering threshold. LABEL will report filtered data as "UNRECOGNIZABLE" within the clade field.

Let P be the set of positive samples appropriate to the module of interest (say, H5 hemagglutinin sequences for module "H5v2011"). We define N to be a set of typical negative samples to be used within a null distribution. Since subtypes H1–H4 and H6–H16 hemagglutinins give us the closest set of samples still not in P, they provide a worst-case scenario for data filtering.

To account for sample variation, we also extract random subsequences from our positive and negative sets at a fixed, minimal expected length of 350 nts (custom Perl script available upon request). These positive and negative sets provide reasonable boundary distributions for length/subsequence sampling variants—denoted S and G respectively. All sets (P, N, S, G) are filtered for non-redundancy.

We establish our threshold for data filtering at the root lineage prediction level where each sequence is analyzed by M profile hidden Markov models using the reverse-corrected score. Let the form $X_i^{(m)}$ denote some sample i from dataset X with a pHMM score belonging to m; the sequence of samples $i, 1 \leq i \leq num(X)$ is ordered from least to greatest by the normalized minimum value. The filter threshold is found by:

$$T_1 = mid\left(\max_i \min_{m \in M} \frac{P_i^{(m)}}{\min[L_i, L_0]}, \min_j \min_{m \in M} \frac{N_j^{(m)}}{\min[L_j, L_0]}\right)$$

$$T_2 = mid\left(\min_{m \in M} \frac{G_k^{(m)}}{\min[L_k, L_0]}, \min_{m \in M} \frac{G_{k+1}^{(m)}}{\min[L_{k+1}, L_0]}\right), \text{where} \frac{k}{num(G)} < 0.001$$

$$\text{Filter Threshold} = \min(T_1, T_2)$$

The function $mid(\cdot)$ is the midpoint between the two data points, the function $num(\cdot)$ is the number of samples in the set, and L_0 is the maximum expected sequence length (prevents long UTRs/etc. from excluding legitimate sequences, we use 2000 nt length for all HA).

For our H5 module we derived P and S from 3,011 non-redundant H5 hemagglutinins shown in Table 1. Sets N and G were derived from 6,842 non-redundant, non-laboratory hemagglutinin sequences submitted to GenBank in 2011 with subtypes H1–H4 and H6–H16 (GenBank accession numbers available upon request). Figure M1 shows T_2 is more suitable for the filtering threshold. The design of our filtering threshold maintains 100% sensitivity to positive samples while allowing less than 0.001 of small subsequences (worst-case scenario) to admitted.

We also applied our threshold guidelines to our H9 module. Positive datasets were derived from 1,592 non-redundant H9 hemagglutinins shown in Table 4. Negative sets were derived from 6,882 non-redundant, non-laboratory hemagglutinin sequences submitted to GenBank in 2011 with subtypes H1–H8 and H10–H16 (GenBank accessions available upon request). Figure M2 shows greater separation for H9 hemagglutinins than H5 such that T_1 was more appropriate as the filter threshold. All figures in this supplemental file were created in Tableau 8.

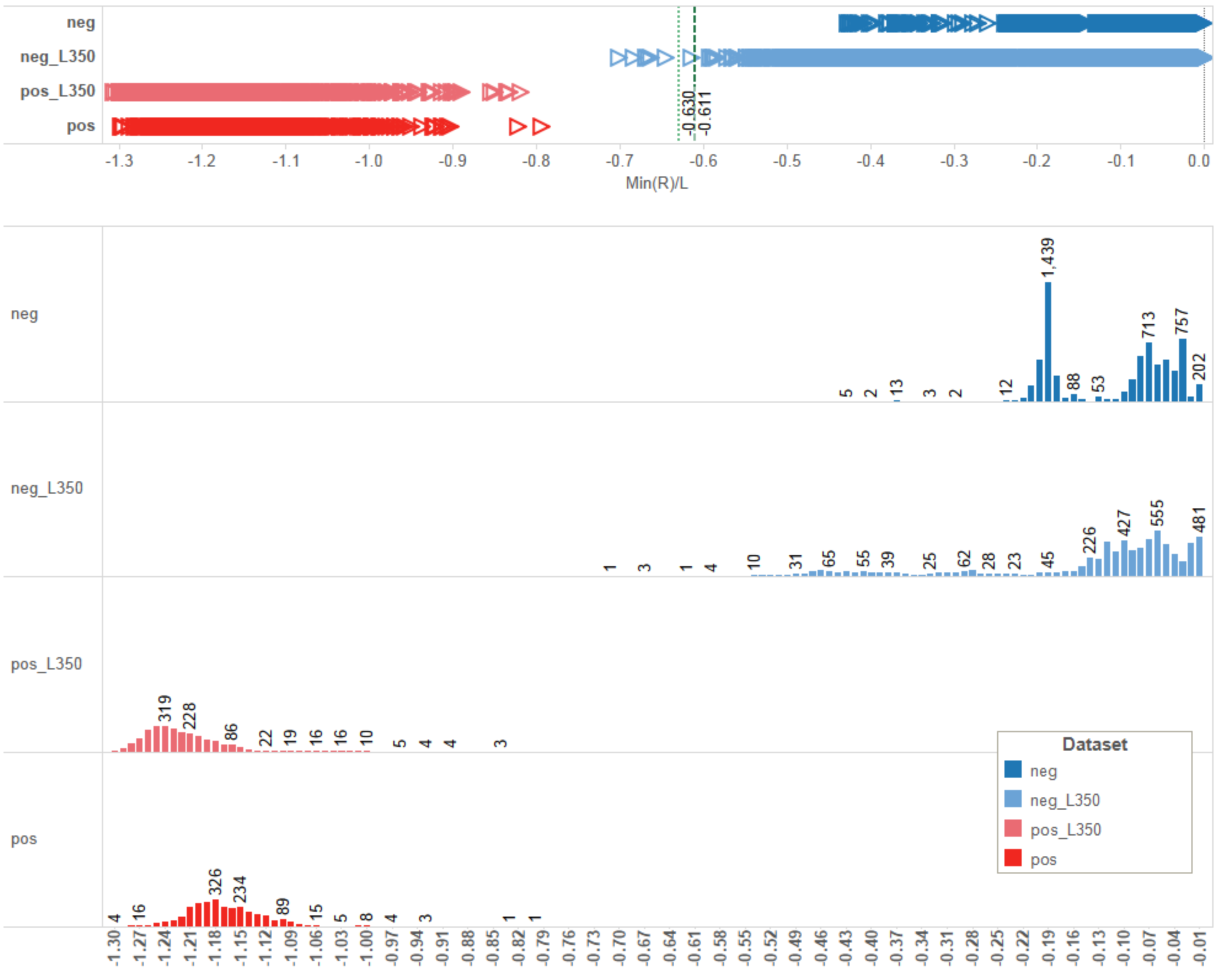

Figure M1: Strip chart and histogram of H5 hemagglutinin positive and negative datasets with 350 nucleotide long subsequence datasets. T_1 and T_2 are shown as light dotted and dark dashed lines respectively.

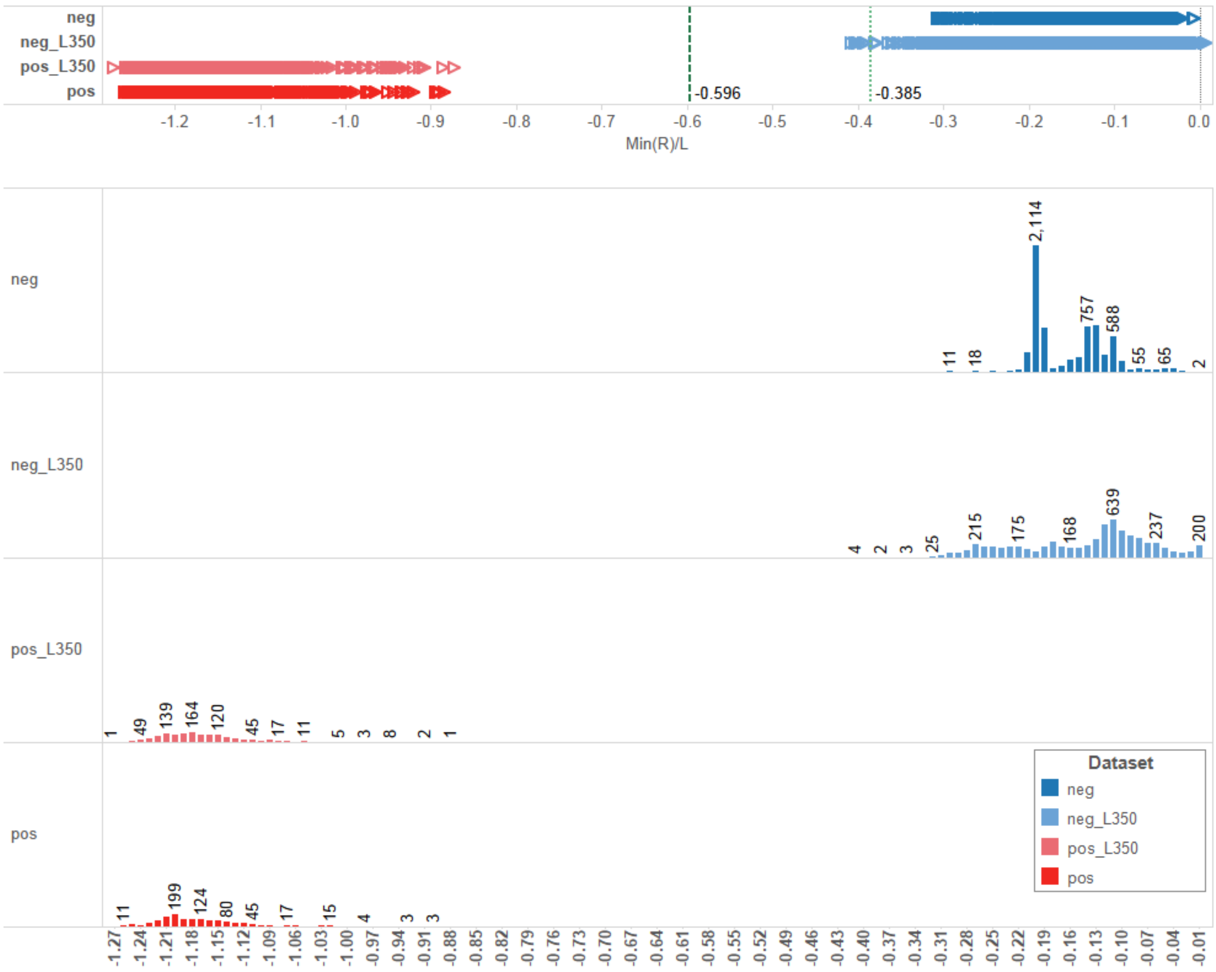

Figure M2: Strip chart and histogram of H9 hemagglutinin positive and negative datasets with 350 nucleotide long subsequence datasets. T_1 and T_2 are shown as light dotted and dark dashed lines respectively.
